# Different Effects of Premature Infant Formula and Breast Milk on Intestinal Microecological Development in Premature Infants

**DOI:** 10.3389/fmicb.2019.03020

**Published:** 2020-01-17

**Authors:** Cheng Chen, Qiuyue Yin, Hui Wu, Lei Cheng, Jung-Il Kwon, Juan Jin, Tongyan Han, Huilian Che

**Affiliations:** ^1^Beijing Advanced Innovation Center for Food Nutrition and Human Health, College of Food Science and Nutritional Engineering, China Agricultural University, Beijing, China; ^2^Department of Neonatology, Nanpi County People’s Hospital, Cangzhou, China; ^3^Department of Neonatology, Peking University Third Hospital, Beijing, China; ^4^Maeil Innovation Center, Maeil Dairies Co., Ltd., Seoul, South Korea

**Keywords:** premature infant, premature infant formula, breast milk, intestinal microecology, environment

## Abstract

Intestinal microecology has been shown to participate in the pathogenesis of many diseases through different pathways, and the intestinal microecology of premature infants is significantly different from full-term infants. Intestinal microecology in premature infants is affected by various factors such as gestational age, diet, antibiotic use. However, there are few studies focus on the effects of diet on intestinal microecological development in premature infants. This study explored the different effects of the formula milk (FM) and breast milk (BM) for the development of intestinal microecology in premature infants. The results showed that BM feeding increases the alpha diversity of the intestinal flora, however, FM feeding contributes to the increase in short-chain fatty acids (SCFAs) in the gut of preterm infants. The growth environment has an important influence on the β diversity of intestinal microecology, the genomic function, and the evolution of intestinal microecology in premature infants. The intestinal microecology in premature infants is significantly associated with gestational age and weight gain. This study explored the effects of feeding methods and growth environment on intestinal microecology in premature infants, and provided a basis for promoting the healthy development of premature infants.

## Introduction

In the past decade, a large number of studies have indicated causal relationship between intestinal microecology and various chronic disease. Human health was affected by intestinal microecology through such as gut-liver axis, gut-lung axis and gut-brain axis (Madan et al., 2012; Nauta et al., 2013). Growing of evidence supports abnormal intestinal microecology associated with the morbidity of type 2 diabetes, obesity, allergy and autism (Kirjavainen et al., 2002; Markestad et al., 2005; Tremaroli and Bäckhed, 2012; Forslund et al., 2015). The developmental period of the gut microbiota is from birth to the 3 years old. The developmental pattern of neonatal intestinal microecology is very complex and is influenced by many factors, including gestational age, environment, birth mode, and feeding method (Muglia and Katz, 2010). Because the developmental pattern of intestinal microecology is complex, it is difficult to define the normal intestinal microecology, but the intestinal microecological development model of exclusively breast-fed infants were considered to be the gold standard (Gartner et al., 2005). Neonatal gastrointestinal microbial colonization begins with facultative anaerobic bacteria, followed by the establishment of anaerobic genus (Aagaard et al., 2014).

Premature infants are a type of infants born under the gestational age of 37 weeks, which have lower weight, lower organ functions than full-term infants. Premature infants are more likely to be born by caesarean section, while full-term infants are more likely to be born through vagina. Due to the fact that caesarean sections are not exposed to microbes of maternal vagina, feces and epithelial, the composition and diversity of their intestinal microecology are significantly different from those of vaginal delivery (Arboleya et al., 2012). Premature infants are usually separated from their mother and exposed to the neonatal intensive care unit (NICU) environment, resulting in delayed colonization of typical symbiotic bacteria such as *Bifidobacteria* based anaerobic bacteria, and are more likely to be colonized by potential pathogenic microorganisms (LaTuga et al., 2011).

Studies on full-term infants have found that different feeding methods are one factor affecting the intestinal microecology (Sherman, 2010). Breastfed infants can gain additional microbiota from breast milk to improve their gut microbiota (Deitch, 2012). Six *Bifidobacteria* have been isolated from breast milk and use in commercial products to improve gut microbiota in infants (Nussbaum and Sperandio, 2011). In addition to probiotics, breast milk (BM) also contains a large number of bifidus factors such as human milk oligosaccharides. Many studies have shown that full-term infants fed with BM have more *Bifidobacteria* and lactic acid bacteria in their intestines (Luciano et al., 2011), while infants fed formula milk (FM) have fewer strict anaerobic bacteria. Other studies have found significant changes in the intestinal microecological development of full-term and premature infants, suggesting that gestational age may be an important factor affecting intestinal microecological development in premature infants (Gidrewicz and Fenton, 2014). However, the effects of different feeding methods on intestinal microecological development in premature infants are still unclear.

Intestinal microecology not only contains gut microbiota, but also a large number of gut microbiota metabolites. SCFAs (mainly acetate, butyrate and propionate) produced by the human gut microbiota ferments dietary fibers, and are important functional substances in the intestinal microenvironment. In addition to decreasing pH in gut lumen and inhibiting the growth of pathogen, SCFAs can regulate immune cell function through cell surface SCFA receptors GPR41, GPR43, and GPR109A and maintain intestinal microecological stability. Acetate, propionate and butyrate play a role in enhancing intestinal barrier and epidemiological studies have found that children with high levels of butyrate and propionate in feces around the age of 1 are less likely to develop asthma and food allergies in the future. Acetate, propionate and butyrate are also the most abundant SCFAs in the intestines, which account for 95% of all short chain fatty acids in gut lumen.

The weight gain of premature infants is an indicator of the overall development of premature infants. The weight is positively correlated with the survival rate of premature infants. Clinical trials have found that infants intestinal microecology affects infant growth and development (Gough et al., 2015) and has been validated by stool transplantation in germ-free animals (Blanton et al., 2016). Although the relevant mechanisms are still unclear, it is speculated that the disorder of growth hormone secretion may be caused by the disorder of intestinal microecology. However, it is not known whether the growth of premature infants’ weight is affected by the gut microbiota.

This study used a longitudinal prospective study and analyzed the fecal samples of premature infants by gene sequencing and gas chromatography. The effects of FM and BM for premature infants intestinal microecological development was compared, and the relationship between gut microbiota and development in premature infants was explored.

## Materials and Methods

### Patient Enrollment and Sample Collection

All samples were collected at Peking The Third Hospital and People’s Hospital of Nanpi Country, and approved by the Human Research Protection Office (approval number IRB00006761-M2017340). Samples were obtained from infants hospitalized in the Neonatal Intensive Care Unit (NICU). Infants were enrolled after parents provided informed consent.

Inclusion criteria: (1) preterm infants born at ≤36 weeks of gestation or birth weight ≤2,500 g, (2) Need to be hospitalized in neonatal intensive care unit (NICU), (3) No congenital malformations.

Non-inclusion criteria: (1) Premature infant born with severe congenital malformations, (2) Survival time less than 7 days.

Standardized feeding guidelines were used, all infants started trophic enteral feeding within 36 h without any special situation and gradually increased the proportion of enteral nutrition to complete enteral feeding. All premature infants were treated according to clinical medical practice for protective ventilation strategies, nutritional support, prevention infections. The general situation of the parents and medical history of the mother during pregnancy was recorded. Because their mothers had almost no breast milk at birth, the preterm infant formula is used for initial enteral feeding. When the enteral feeding was increased as tolerated by 15 ml per day, the breast milk was replaced with enteral feeding. All premature infants were given priority in breastfeeding, formula was used when breastfeeding was not available. According to the proportion of oral intake of breast milk, more than 50% was classified as breast milk group; and the others was classified into formula milk group.

To the day of birth as day 0, collected stool samples of premature infants at day 0, 7, 14, 21, 28. Blood biochemistry and blood routine testing were performed weekly during hospitalization, and commonly used clinical nutrition indicators (total protein TP, albumin ALB, Hemoglobin HGB, calcium CA, total bilirubin TBIL, direct bilirubin DBIL, blood urea nitrogen BUN, uric acid, URIC) were selected for analyzed.

All premature infants needed to change their diapers every 3 h, when changed diapers, collected the stool samples from the diapers, and stored the stool samples in the frozen tubes, at −80°C until processed. Blood samples were taken to the hospital laboratory for testing immediately after they were obtained.

### Short Chain Fatty Acid Extraction Procedure

Fecal samples were weighed and suspended in 1 mL of water with 9% of formic acid per 0.1 g, and homogenized with a vortex for 2 min and centrifuged for 15 min at 12000 × *g*. Each milliliter of water supernatant was extracted with 1 mL of diethyl ether for 8 h at 4°C, and centrifuged for 10 min at 12000 × *g*. Diethyl ether extracts was transferred into a sample bottle.

### GC Analysis

Quantitative determination of SCFA samples was performed by GC using the gas chromatograph Agilent 7890 C (Agilent Technologies Inc. United States) equipped with a flame ionization detector (FID) and an N10149 automatic liquid sampler (Agilent, United States). For separation, a packed column with Free Fatty Acid Phase (DB-FFAP, Agilent Technologies Inc., United States) of 30 m × 0.53 mm I.D. coated with 0.50 μm film thickness was used. N2 as carrier gas were used under isothermic conditions at 130°C (injector at 225°C; detector at 250°C). Each sample measurement (volume: 1 μl) was initiated by a calibration with SCFA standards in the respective buffer solutions.

### Gut Microbiome Measurement

DNA extraction. Stool samples from were extracted using a cetyltrimethylammonium bromide (CTAB)-buffer-based protocol, followed by detected the concentration of DNA by agarose e-gel, and dilute the sample to 1 ng/μl with sterile water. Diluted DNA was used as template, and the V4 region of the bacterial 16S rRNA was sequenced with the 515F and 806R. Amplicons were pooled and verified using a 2% TBE agarose e-gel, after quality check, quantification and purification. Samples were sequenced on the Thermofisher Ion S5^TM^XL sequencer as a service by the Beijing Novogene Technology Co., Ltd.

Cutadapt (version 1.9.1) was used to remove the low quality part of the reads, and then separate the sample data from the obtained reads according to Barcode, and cut off the initial data of Barcode and primer sequences to get the raw data (Raw reads). The raw Reads sequence were checked for chimeras using UCHIME and filtered from the data set to obtain Clean Reads. Clean Reads were clustered to operational taxonomic units (OTUs) *de novo* with 97% sequence identification. Taxonomy was assigned using the classifier and SILVA132 reference database.

Alpha diversity indicators, including Shannon index, Chao 1 index and Pielou index were calculated with R (version 3.6.0) package “vegan” (version 2.5-6). Beta diversity indicators, including weighted Unifrac principle coordination analysis (PCoA) and Non-metric multidimensional scaling (NMDS) analysis were also calculated with R package “vegan” (version 2.5-6). The Venn plots of two groups in OTUs as well as in family taxa were plotted with R package “VennDiagram” (version 1.6.20). Canonical Correlation Analysis (CCA) was performed with R package “vegan” (version 2.5-6). Genomic function was predicted by R package “Tax4Fun” (version 0.3.1). Except for the Venn figures, all other figures were plotted with R package “ggplot2” (version 3.1.1). Bacteria with abundance greater than 1% in any sample was selected for Linear discriminant analysis Effect Size (LEfSe), and a total of 97 bacteria were selected. LEfSe analysis was performed by “Galaxy/Hutlab^1^ “using default values (alpha value 0.5 and threshold 2.0 for logarithmic linear discriminant analysis score for discriminative features) and the strategy for multi-class analysis set to “all-against-all.”

### Statistical Analyses

Depending on the data distribution, Student’s *t*-test (unpaired, two tailed) or a Mann Whitney test was used to calculate significance levels between treatment groups. *P* < 0.05 was considered significant. Graph generation and statistical analyses were performed using R version 3.6.0.

## Results

A total of 60 premature infants were enrolled in the study from October 2017 to October 2018. All individuals included in our study were born premature (<36 weeks) and 9 individuals had very low birth weight (VLBW; <1,500 g). The majority of infants were delivered by cesarean-section, born after 32 gestational age and with the birth weight between 1500 and 2500 g. Enteral feedings were introduced on the postnatal day 2. The infant feeding type was categorized based on the BM and FM fed proportions. 30 premature infants were included in the BM group as they received breast milk more than 50% of feedings, and the remaining 30 premature infants were included in the FM group. A total of 316 stool samples were collected longitudinally from 60 premature infants during their NICU hospitalization. The table containing clinical information for each infant was summarized in Table 1. Two groups had similar baseline characteristics and had no significant difference in gestational age, birth weight, birth mode, multiple birth, gender, and antibiotic use.

**TABLE 1:** Basic information of preterm infants.

	**Breast milk group (*n* = 30)**	**Formula group (*n* = 30)**
**Gestational age**		
<28	5	4
28∼32	9	6
≥32	16	20
**Birth weight**		
<1000	5	4
1000∼1500	8	5
1500∼2500	13	19
≥2500	4	2
**Gender**		
Male	17	14
Female	13	16
**Multiple birth**		
Yes	9	10
No	21	20
**Birth mode**		
Cesarean	21	24
vagina	9	6
**Apgar Score at 1 min**		
1–3	0	2
4–6	5	4
7–9	11	16
10	14	8
**Antibiotic in 48 h**		
Yes	21	23
No	9	7

### Clinical Indicators of Preterm Infant

The main clinical indicators of all preterm infants during hospitalization are shown in Table 2. The weight gain of preterm infants in the FM group with gestational age less than 32 weeks was significantly higher than that in BM group. In the second week after birth, the level of bilirubin in FM preterm infants was significantly lower than that in BM group. Except for changes in body weight and total bilirubin, there were no significant differences between the two groups in preterm infants.

**TABLE 2:** Preterm infant clinical indicators.

**Clinical indicators**	**FM group**		**BM group**	
	14.4 ± 7.3		12.8 ± 8.2	
**Weight Gain**	**≤32 weeks**	**>32 weeks**	**≤32 weeks**	**>32 weeks**
	14.6 ± 6.9^#^	14.2 ± 7.6	9.8 ± 2.7^#^	13.8 ± 9.0

	**Week 1**	**Week 2**	**Week 1**	**Week 2**

TP	47.2 ± 4.7	46.1 ± 5.0	48.8 ± 4.1	48.4 ± 5.0
ALB	31.4 ± 2.2	30.6 ± 2.9	33.0 ± 2.3	32.2 ± 3.1
HGB	194.3 ± 16.6	160.5 ± 18.8	191.7 ± 20.5	163.0 ± 24.9
CA	1.98 ± 0.17	2.27 ± 0.25	1.94 ± 0.18	2.17 ± 0.16
TBIL	87.7 ± 22.0	117.7 ± 31.1^∗^	88.1 ± 22.7	136.8 ± 28.7^∗^
DBIL	8.3 ± 3.9	11.8 ± 4.7	9.7 ± 2.2	16.7 ± 5.7
BUN	5.3 ± 1.8	3.2 ± 1.5	5.5 ± 2.0	3.5 ± 1.7
URIC	479.5 ± 132.9	170.9 ± 90.4	471.0 ± 105.7	173.7 ± 74.6

*Significant differences between the two groups of the same symbol (^∗^, #), *p* < 0.05.*

### Short Chain Fatty Acids (SCFAs)

**FIGURE 1:**
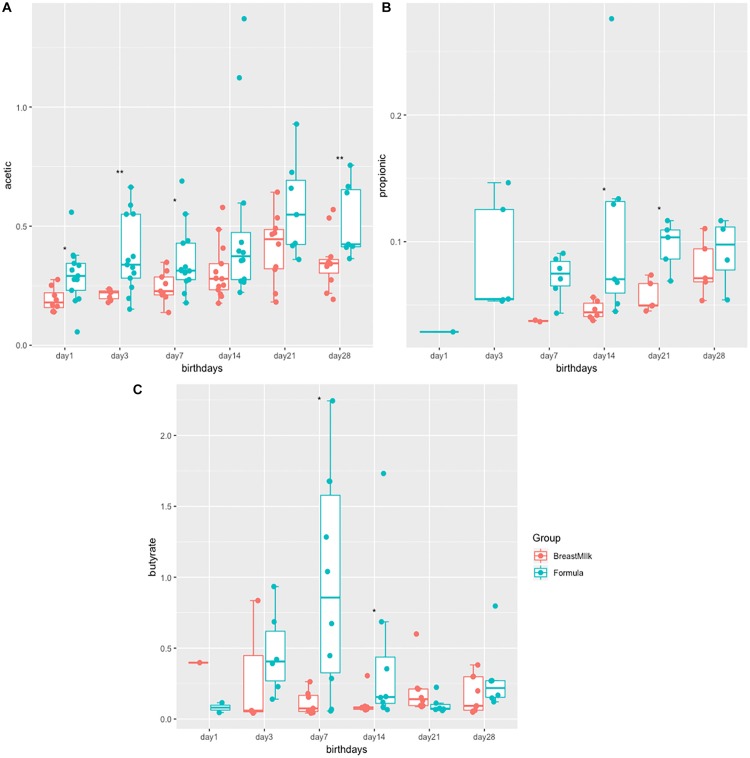
SCFA levels in preterm infants in FM and BM groups. **(A)** Concentration of acetate in stool samples. **(B)** Concentration of propionate in stool samples. **(C)** Concentration of butyrate in stool samples. ^∗^*p* < 0.05, ^∗∗^*p* < 0.01.

The study examined the concentrations of acetic acid, propionate and butyrate in all 316 stool samples of two group premature infants. In both groups, acetate and butyric acid are the two most abundant SCFAs in the intestine (Figures 1A,C). The concentration of acetate was the highest in three SCFAs, and gradually increases with birth time. The acetate concentrations in the FM group was significantly higher than that in the BM group after birth (Figure 1A). The concentration of propionate was the lowest in three SCFAs (Figure 1B), and produced later in BM group. Propionate content in FM group showed a fluctuating trend, however in BM group, showed a gradual increase trend. On the day 28 after birth, no significant difference in propionate between BM group and FM group. Butyrate was the most susceptible SCFA in intestinal microecology of premature infants and showed a trend of fluctuation. On the day 7, the content of butyrate in the feces of FM group was significantly higher, however by 21 days after birth, the concentration of butyrate in the BM group was significantly higher than that in the FM group. In general, the short-chain fatty acid levels in FM group were higher than those in BM group, and the trend was more intense in FM group than in BM group.

### Composition of Intestinal Flora in Premature Infants

**FIGURE 2:**
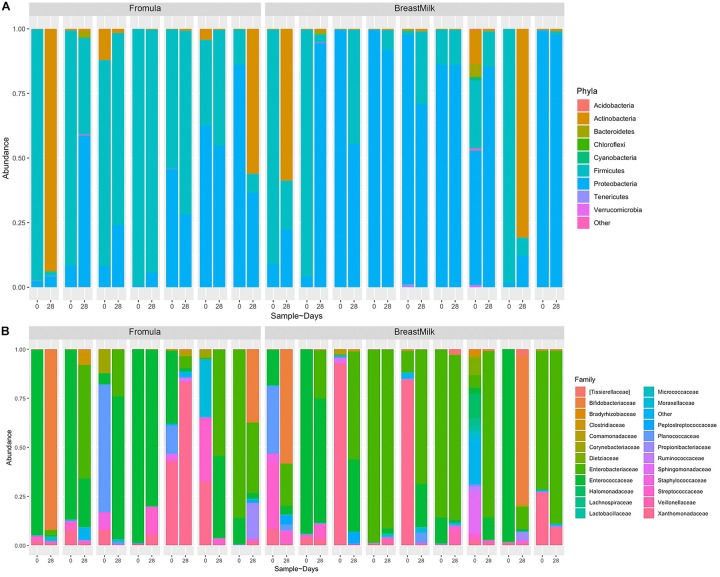
Composition of gut microbiota in premature infants at phylum and family levels. **(A)** Nine most abundant bacteria at the phyla level. **(B)** Twenty three most abundant bacteria at the family level.

Due to the gut microbiota was influenced by birth mode and gestational age, we selected stool samples from 17 premature infants with same gestational age (32 weeks) and birth mode (caesarean section) for 16S rDNA analysis. Because DNA extracted from one fecal sample did not meet quality standards, the two samples of this infant was excluded. Finally, total 32 fecal samples from 16 premature infants were analyzed by 16S rRNA gene sequencing. From the Phylum level analysis (Figure 2A), the fecal components of all premature infants are mainly composed of *Firmicutes*, *Proteobacteria*, and *Actinobacteria*, and account for more than 99% of all microorganisms. However, the proportion of *Bacteroides*, which is an important component in full term infants, is very small, accounting for only 0.3% of the total microbial composition. From the level of the family (Figure 2B), the intestinal flora of premature infants is mainly composed of *Enterobacteriaceae*, *Enterococcaceae*, *Xanthomonadaceae*, *Bifidobacteriaceae*, *Planococcaceae*, *Streptococcaceae*, *Moraxellaceae*, which accounts for more than 90% of all microorganisms.

### Intestinal Flora Alpha Diversity in Premature Infants

**FIGURE 3:**
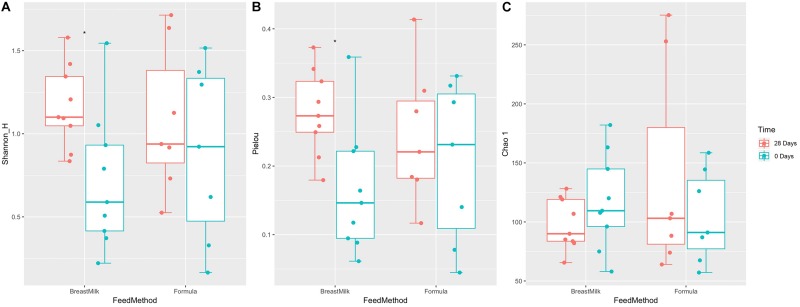
Gut microbiota alpha diversity in two preterm infant groups. Shannon index **(A)**, Pielou index **(B)**, and Chao.1 index **(C)** before and after feeding for 28 days in preterm infants with different feeding methods. ^∗^*p* < 0.05.

The gut microbiota alpha diversity of the two premature infant groups was evaluated by Shannon index, Chao1 index and Equitability index (Figure 3). We first compared the difference in gut microbiota between the two preterm infant groups at 0 and 28 days of birth. The Shannon index and Equitability index of intestinal flora in BM group increased significantly (Figures 3A,B), however there was no significant difference in FM group. And there was no significant difference in the Chao1 index between the two groups at day 0 and day 28 (Figure 3C). However, when we compared the gut microbiota alpha diversity of premature infants in BM group and FM group, no significant differences were found in the three indexes, either in the day 0 samples or day 28 samples. The results indicate that breastfeeding increases the alpha diversity of the intestinal flora in preterm infants, while milk powder feeding and the environment do not affect the alpha diversity of intestinal microecology in preterm infants. And different feeding methods have no significant effect on the alpha diversity of gut microbiota in premature infants.

### Intestinal Flora Beta Diversity in Premature Infants

**FIGURE 4:**
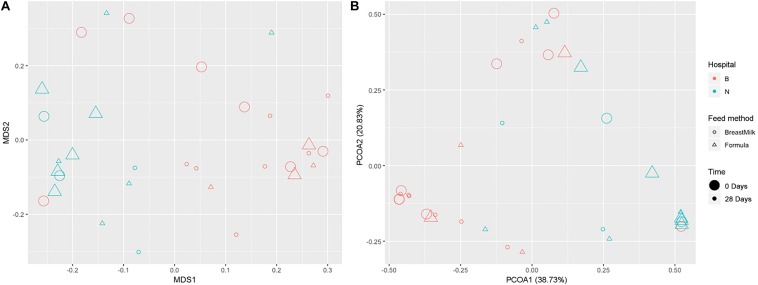
Gut microbiota beta diversity in preterm infants. NMDS **(A)** and PCoA **(B)** analysis grouped by feeding method, time, and environment.

We used two analytical methods, NMDS analysis (Figure 4A) and PCoA analysis (Figure 4B), to compare beta diversity in preterm infants. According to the feeding method, hospital environment and feeding time, it was found that in the two analytical methods, the samples were clearly classified according to the hospital environment. No significant separation of samples according to feeding time and feeding method was found in the same hospital environment. The result suggesting that the growth environment has a significant effect on the beta diversity of intestinal microecology in preterm infants during the first month of life, while the feeding method had no significant effect on the β diversity of intestinal microecology in premature infants.

### Succession of Intestinal Microecology in Premature Infants

**FIGURE 5:**
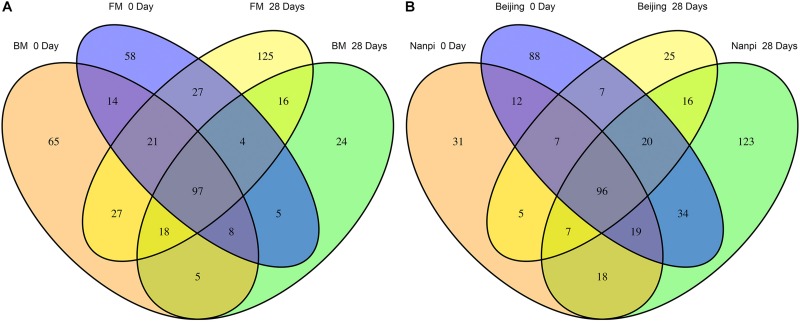
Differences in gut microbiota before and after feeding for 28 days. **(A)** Effects of different feeding methods on the development of gut microbiota in premature infants. **(B)** Effects of different environment on the development of gut microbiota in premature infants.

We first compared the community changes of intestinal flora in preterm infants between 0 and 28 days after birth according to different feeding methods (Figure 5A). The results showed that after 28 days of BM feeding, the species of preterm infant intestinal flora decreased from 255 (100%) at birth to 177 (69.4%), of which 49 (19.2%) were newly obtained, and 127 (49.8%) were lost. The main acquired species were *Firmicutes* (10 species), *Proteobacteria* (9 species) and *Actinobacteria* (5 species), and the main lost species were *Proteobacteria* (47 species), *Actinobacteria* (20 species), *Firmicutes* (15 species) and *Bacteroides* (14 species). After 28 days of FM feeding, the species of preterm infant intestinal flora 234 (100%) at birth to 335 (152%), of which 186 (79.5%) were newly obtained, and 85 (36.3%) were lost; The main acquired species were *Proteobacteria* (55 species), *Firmicutes* (54 species), *Bacteroidetes* (22 species) and *Actinobacteria* (20 species), and the main lost species were *Proteobacteria* (37 species) and *Actinobacteria* (14 species).

According to the comparison of different hospital environments (Figure 5B), after 28 days of feeding in Beijing hospital, the species of preterm infant intestinal flora decreased from 283 (100%) at birth to 183 (64.7%), of which 53 (18.7%) were newly acquired, 153 (54.1%) species were lost. The main lost species were *Proteobacteria* (59 species), *Actinobacteria* (31 species), *Bacteroidetes* (14 species) and *Firmicutes* (13 species). The main obtained species were *Firmicutes* (19 Species), *Proteobacteria* (15 species) and *Actinobacteria* (10 species); after 28 days of feeding in Nanpi hospital, the species of preterm infant intestinal flora increased from 195 (100%) at birth to 333 (171%). Among them, 55 (28.2%) species were lost, and 193 (99.0%) species were newly obtained. The main lost species were *Proteobacteria* (23 species), *Firmicutes* (8 species) and *Actinobacteria* (8 species). The main obtained species were *Proteobacteria (63 spec*ies), *Firmicutes* (55 species) and *Bacteroides* (19 species) and *Actinobacteria* (19 species).

Since the results of grouping according to feeding methods and environment are similar, considering that it may be due to interaction, we further used two-way ANOVA to explore the interaction between the environment and the feeding method (Table 3). The results showed that the interaction was not significant (the *P* values of obtained species and lost species were 0.491 and 0.584, respectively). After eliminating the interaction effect, two-way ANOVA analysis was performed again. The results showed that the hospital environment significantly affected the number of obtained species (*P* = 0.033), while the feeding method did not have a significant effect (*P* = 0.288). The impact of the hospital environment and feeding methods on lost species was not significant.

**TABLE 3:** Numbers of obtained and lost bacteria in premature infants.

	**Obtained species**		**Lost species**	
	**FM**	**BM**	**FM**	**BM**
Beijing	24.5 ± 8.5	29.7 ± 6.7	51.5 ± 27.5	45.5 ± 26.7
Nanpi	74.5 ± 48.5	25.0 ± 1.0	27.2 ± 11.8	22.5 ± 6.5

### Biomarkers for Different Groups of Preterm Infants

**FIGURE 6:**
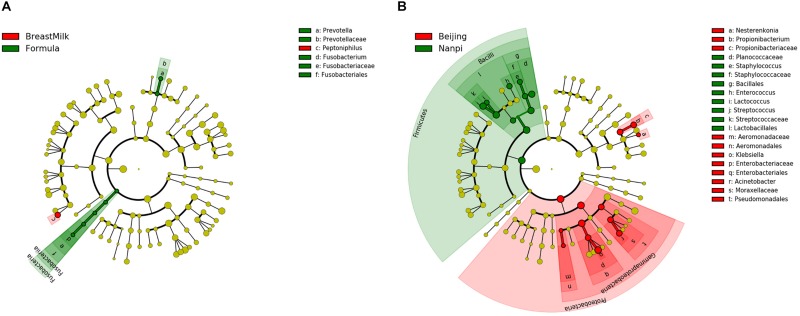
Differentially abundant microbial clades in premature infant stool samples. **(A)** LEfSe analysis of gut microbiota in different feeding methods. **(B)** LEfSe analysis of gut microbiota in different feeding environment.

Linear discriminant analysis effect size (LEfSe) analysis was carried out to determine which bacteria were driving the differences between two groups. First, the different biomarkers between FM group and BM group were analyzed (Figure 6A). These analyses showed that two bacteria (*Fusobacterium* and *Prevotella copri*) were present within the FM group samples that were either not present or in much lower amounts in the BM group samples. A further *Peptoniphilus* was present in higher amounts in the BM group samples compared to FM group samples. Because we found that the environment has a significant impact on the composition of the gut microbiota, later, we compared biomarkers in different environments (Figure 6B). *Firmicutes* were present in higher amounts in Nanpi hospital groups, however, *Proteobacteria* were present in higher amounts in Beijing hospital groups.

### Correlation Analysis of Intestinal Microenvironment and Environmental Factors and Prediction of Intestinal Flora Function

**FIGURE 7:**
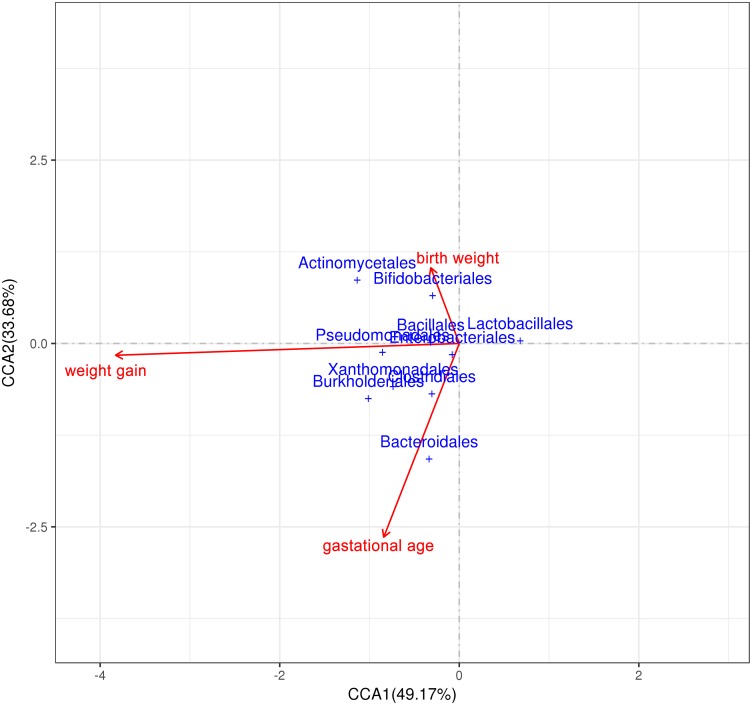
Correlation analysis of intestinal microenvironment and environmental factors. Correlation analysis between environmental factors (birth weight, weight gain and gestational age) and gut microbiota.

The correlation of three environmental factors (gestational age, birth weight, and average daily weight gain) and gut microbiota was analyzed using Canonical Correlation Analysis (CCA) (Figure 7). In the top 10 species with the highest abundance, the abundance of *Bifidobacterium* and *Actinomycetes* was positively correlated with birth weight; and the abundance of *Bacteroides* was positively correlated with the gestational age. There was a weak positive correlation between *Actinomycetes*, *Pseudomonas aeruginosa*, *Burkholders* and the weight gain premature infants.

**FIGURE 8:**
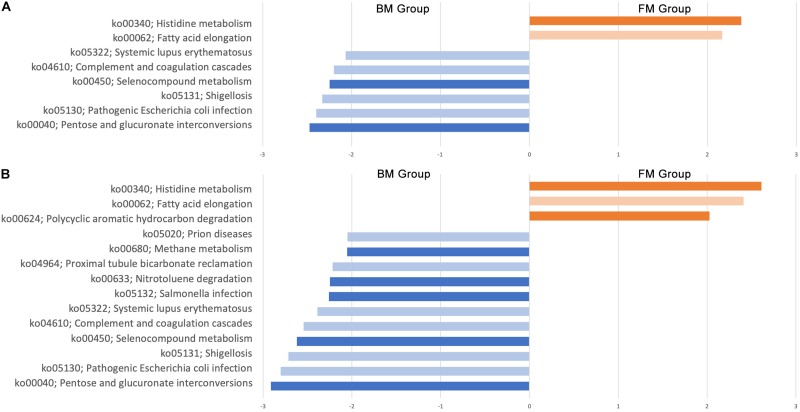
Genomic differential signaling pathway in gut microbiota of premature infants. **(A)** Effect of feeding methods on gut microbiota function. **(B)** Effect of environment on gut microbiota function. Dark color indicates the abundance greater than 0.1%, Light color indicates abundance less than 0.1%.

The function of the intestinal flora of premature infants was predicted by the Tax4Fun package in R. A total of 275 signal pathways were included in all samples. First, we compared the function of the gut microbiota in BM group and FM group at day 0 and day 28. On day 0, there was no significant difference in gut microbiota function between the two groups in all 275 signaling pathways. However, there were significant differences in the 8 signaling pathways after 28 days (Figure 8A), of which 3 signaling pathways are effect (abundance bigger than 0.1%). The pathways of pentose and glucuronate interconversions (ko00040) and selenocompound metabolism (ko00450) were higher in BM group, however, histidine metabolism (ko00340) was higher in FM group. Considering that the environment also has an impact on the gut microbiota, we continue to explore the effects of the environment on the function of the gut microbiota in subgroups. After sub-grouping by environment, 6 more signaling pathways were found to have significant differences (Figure 8B), of which 7 are effective (abundance bigger than 0.1%). The results show that both feeding methods and the environment affect the function of the gut genome.

## Discussion

In the clinical growth and nutritional indicators of the two groups of premature infants, only the different feeding methods in the preterm infants with gestational age less than 32 weeks showed significant differences in weight gain. This was consistent with previous studies showing that formula milk has the effect of promoting weight gain (Elizabeth et al., 2013), probably due to the tolerance, high nutrient and high energy content of milk powder. The energy content of the 16% concentration premature infant formula used in this clinical trial was 81 kcal/100mL, which was higher than BM (60∼77 kcal/100 mL) (Gidrewicz and Fenton, 2014).

There are differences in concentrations of SCFAs between two premature infant groups. The concentrations of acetate and butyrate in formula group were significantly higher than those in BM group, which was consistent with the higher abundance of *Firmicutes* in the feces. The short-chain fatty acids in the feces are mainly produced by the fermentation of dietary fibers through the gut microbiota (Koh et al., 2016), while the FM does not contain dietary fiber or prebiotics. It is speculated that the SCFAs in the formula group may not be produced by the classical pathway of fermenting dietary fiber, which may be caused by the decomposition of amino acids by the gut microbiota (Repa et al., 2015). SCFAs are generally considered to be a class of beneficial substances (Koh et al., 2016), especially butyrate (Luying et al., 2009), which can enhance the intestinal epithelial barrier and regulate immune function. However, previous studies have found that the content of butyrate was higher in the intestines of food-allergic infants (Canani et al., 2016). Further researches are needed to explore the relationship between short-chain fatty acid content and infant health.

The results of gut microbiota showed that premature infants in two groups had significant differences in Phylum level. The relative abundance of *Firmicutes* has been considered to be associated with host weight gain, the increase in body weight is accompanied by an increase in the abundance of *Firmicutes* (Koliada et al., 2017). Abundance of *Firmicutes* in FM group was higher than in BM group, which may be the reason that the weight gain of premature infants in the FM group was faster than BM group. Previous studies have found that the most significant factor affecting intestinal microecology in premature infants is gestational age (Arboleya et al., 2015; Dogra et al., 2015), but in this study it was not found that gestational age has a significant effect on the gut microbiota. Since two research centers were selected for this study, we found that the gut microbiota of premature infants in different research centers has significant differences and can be well distinguished, indicating that the intestinal microecology of premature infants is significantly affected by the environment. It is also the first time found that the gut microbiota of premature infants is mainly affected by the environment. The diversity of gut microbiota in premature infants of BM group showed an increasing trend, while the diversity of gut microbiota in FM group premature infants was almost unchanged, which was not completely consistent with the previous research results (Cong et al., 2017). Premature infants have a higher probability of developing lung infections after birth and need to use antibiotics intravenously to prevent infection. The unchanged gut microbiota diversity in FM group may be caused by the use of antibiotics (Arboleya et al., 2015), and the BM contains prebiotics such as human milk oligosaccharides (Zivkovic et al., 2011), which have a certain protective effect on the diversity of gut microbiota.

The LEfSe analysis found that premature infants in BM group and the FM group had only significant differences among the three bacteria. *Fusobacterium* is an anaerobic Gram-negative pathogen that causes appendicitis in children (Rogers et al., 2016) and is found in murine model to result in premature (Han et al., 2004). Bacteria from the *Prevotella* family had been found to be higher in formula fed infants than breast fed infants (Holgerson et al., 2013). In this study, we also found the same result, *Prevotella* family could be used as a biomarker to distinguish different feeding methods. Unlike previous studies, we did not find that *Lactobacillus* have a higher level in breast fed premature infants, which may be due to the difficulty in colonization of *Lactobacillus* caused by immature intestinal in premature infants (Collado et al., 2015; Dogra et al., 2015).

Canonical Correlation Analysis was used to analysis environment factors such as gestational age, birth weight, and postnatal weight gain, it was found that there was the strongest correlation between weight gain and gut microbiota composition, indicating that the composition of the gut microbiota significantly affects the weight gain of premature infants (Underwood et al., 2009). The *Actinomycetes*, *Pseudomonas aeruginosa* and *Burkholders* showed a weak positive correlation with the weight gain of premature infants. Because weight gain is the result of a combination of enteral and parenteral nutrition, parenteral nutrition in the premature infants early nutritional support strategy occupies a higher proportion, which may be the reason for not finding strong related species. It is also possible that the synergy between multiple species affects the growth and development of premature infants.

Gene function predictions suggest that both feeding methods and environment affect the function of the gut microbiota, however, feeding methods mainly affect the signaling pathways of energy and nutrient metabolism, and the environment affects other signaling pathways. Differences in methane metabolic pathways may be due to endogenous differences in gut microbiota (Pimentel et al., 2013). And the difference in expression of the polycyclic aromatic hydrocarbon degradation, salmonella infection and nitrotoluene degradation signaling pathways may be due to the presence of the contaminants in the environment. Since we did not sample the environment, we cannot confirm this assumption. This suggests that we need to pay attention to the impact of environmental factors on gut microbiota in subsequent studies.

## Conclusion

**FIGURE 9:**
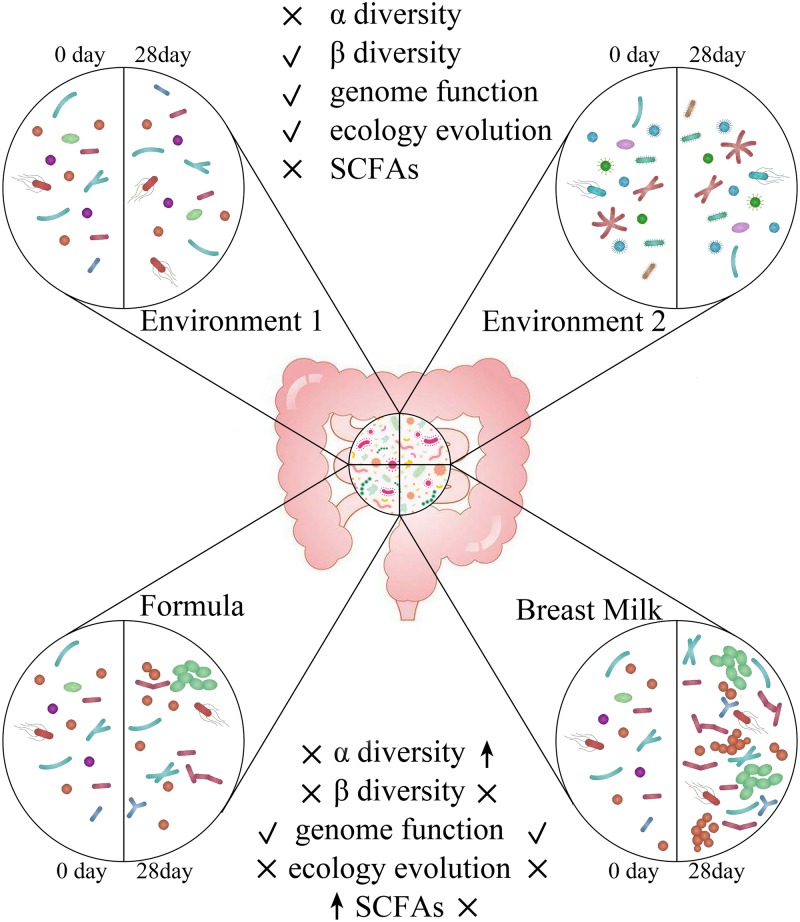
Different effects of environment and feeding methods on gut microbiota in premature infants. ↑indicates will increase the indicator, √indicates has an influence on the indicator, × indicates has no influence on the indicator.

In summary, we found that feeding methods significantly affect the content of SCFAs, the alpha diversity and the function of the gut microbiota in the stool samples of premature infants. Breast feeding helps to promote the increase in alpha diversity and the metabolic function of energy and nutrients in the gut microbiota of premature infants (Figure 9). However, formula feeding increases the concentration of SCFAs in the intestine and the amino acid metabolism of the gut microbiota. The difference in gut microbiota between BM group and FM group is mainly Fusobacteria. In our research, environmental factors were found to have an important impact on the gut microbiota. Which can affect the beta diversity, ecological succession, and microbiota genome functions.

## Data Availability Statement

The raw data sets generated in this study can be found in the ENA database, Primary Accession number PRJEB34505, and Secondary accession number ERP117416.

## Ethics Statement

The studies involving human participants were reviewed and approved by Peking University Third Hospital Medical Science Research Ethics Committee. Written informed consent to participate in this study was provided by the participants’ legal guardian/next of kin.

## Author Contributions

HC supervised and conceptualized the study. CC worked on the methodology and data analysis. QY, HW, LC, and TH were responsible for data curation. J-IK and JJ worked on the formal analysis.

## Conflict of Interest

J-IK and JJ were employed by the Maeil Innovation Center belongs Maeil Dairies Co., Ltd. The remaining authors declare that the research was conducted in the absence of any commercial or financial relationships that could be construed as a potential conflict of interest. The reviewer JW declared a shared affiliation, with no collaboration, with several of the authors, CC, LC, and HC, to the handling Editor at the time of review.

## Footnotes

1 http://huttenhower.sph.harvard.edu/galaxy
